# Intravenous cyclophosphamide induces remission in children with difficult to treat steroid resistant nephrotic syndrome from minimal change disease

**DOI:** 10.1186/s12882-021-02605-6

**Published:** 2021-11-29

**Authors:** Maha Haddad, Arundhati Kale, Lavjay Butani

**Affiliations:** Section of Pediatric Nephrology, University of California Davis, 2516 Stockton Blvd, Sacramento, CA 95817 USA

**Keywords:** Steroid resistant nephrotic syndrome, Cyclophosphamide, Pediatric, Remission

## Abstract

**Background:**

Steroid resistant nephrotic syndrome (SRNS), while uncommon in children, is associated with significant morbidity. Calcineurin inhibitors (CNIs) remain the first line recommended therapy for children with non-genetic forms of SRNS, but some children fail to respond to them. Intravenous (IV) cyclophosphamide (CTX) has been shown to be effective in Asian-Indian children with difficult to treat SRNS (SRNS-DTT). Our study evaluated the outcome of IV CTX treatment in North American children with SRNS-DTT.

**Methods:**

Retrospective review of the medical records of children with SRNS-DTT treated with IV CTX from January 2000 to July 2019 at our center. Data abstracted included demographics, histopathology on renal biopsy, prior and concomitant use of other immunosuppressive agents and serial clinical/laboratory data. Primary outcome measure was attainment of complete remission (CR).

**Results:**

Eight children with SRNS-DTT received monthly doses (median 6; range 4–6) of IV CTX. Four (50%) went into CR, 1 achieved partial remission and 3 did not respond. Three of the 4 responders had minimal change disease (MCD). Excluding the 1 child who responded after the 4th infusion, the median time to CR was 6.5 (range 0.5–8) months after completion of IV CTX infusions. Three remain in CR at a median of 8.5 years (range: 3.7–10.5 years) after completion of CTX; one child relapsed and became steroid-dependent. No infections or life-threatening complications related to IV CTX were observed.

**Conclusions:**

IV CXT can induce long term remission in North-American children with MCD who have SRNS-DTT.

## Background

While children with steroid resistant nephrotic syndrome (SRNS) comprise a small fraction of all children with nephrotic syndrome (NS) [1], such children experience significant morbidity from their underlying disease and are at risk of progression to end stage renal disease (ESRD) [2]. Many studies have demonstrated the efficacy of CNIs in inducing remission in children with non-genetic forms of SRNS and their superiority over other agents such as intravenous (IV) cyclophosphamide (IV CTX) such that tacrolimus and cyclosporine have become first line therapies for children with SRNS [3]. However, not all children with SRNS are responsive to CNIs [4]; remission rates with CNIs have been reported to range from 50 to 80% [5–7]. These children with difficult to treat SRNS (SRNS-DTT) pose an even greater management challenge to pediatric nephrologists due to their poor response to other, even newer immunosuppressive agents, poor quality of life and high risk of progression to ESRD [8, 9].

Data on children of Indian descent with SRNS treated with IV CTX have shown that a small subset of patients do achieve complete remission (CR), either when IV CTX is used as first line therapy [5, 10, 11] or after failure of a trial of high dose IV steroids [12] and based on these data, IV CTX has been recommended as first line therapy for children with SRNS who are not able to be treated with CNIs [13]. These data have not been replicated in children of other race-ethnicities [11, 14].

The aim of our study was to report our experience with the use of IV CTX in treating North-American children with SRNS-DTT who had failed to respond to tacrolimus. Based on our experience, we offer IV CTX as an alternative strategy for these children, in the hope of preventing progression to ESRD.

## Methods

We conducted a retrospective review of the medical records of children (≤18 years at diagnosis) who were treated at our center with IV CTX for primary SRNS-DTT, between January 2000 to June 2019. The diagnosis of primary SRNS was made based on a lack of response to at least of 6 weeks of daily prednisolone therapy (2 mg/kg/day) and a negative serologic work up for secondary causes (normal complement C3 and C4 and negative antinuclear antibody). All patients received at least a 6-month trial of oral tacrolimus (target trough level 10–15 ng/ml); failure to respond to tacrolimus (even partially) led to the categorization of patients as having SRNS-DTT.

Patients’ records were reviewed and the following data were collected: demographic characteristics, initial or late steroid resistance, histopathologic findings on renal biopsy (including electron microscopy), genetic studies for nephrotic syndrome (for mutations in PLCE1, LAMB2, WT1, NPHS1 and NPHS2 genes), use of other medications prior to and concomitant with CTX, details pertaining to the CTX infusions and hospitalizations or treatment for any complications including infections. Laboratory data collected included serum albumin concentration, spot urine protein/creatinine ratio (Up/c) and serum creatinine concentration. Laboratory data were serially followed, from 3 months prior to the first infusion of CTX until last follow up. Since patients were treated by different pediatric nephrologists during the study period, the specific treatment regimen (and dosing) followed was at the discretion of the treating physician and not protocolized. Some patients received IV methylprednisolone (MP) along with their CTX dose, based on their physician’s preference and discretion. Supportive treatment during the IV CTX infusions was protocolized at our institution and consisted of IV saline infusion (at 1.5 times maintenance rate), antiemetics, mesna and furosemide.

The primary outcome measure was complete remission (CR), which was defined as a Up/c < 0.2 (mg/mg) on at least three consecutive occasions and a normal serum albumin (> 3.5 g/dl). Secondary outcome measures were partial remission (PR), defined as Up/c > 0.2 and ≤ 1.0 (mg/mg) with a serum albumin of 2.5–3.4 g/dl, and time to remission.

The study was approved by the University of California Institutional Review Board; all methods were performed in accordance with the relevant guidelines and regulations. Exemption was granted for obtaining written informed consent from study subjects.

## Results

### Baseline characteristics

During the study period, 8 patients (7 male; 1 female) with DTT-SRNS were treated at our center and all received IV CTX. Two patients were Caucasian, 4 were Latino, 1 was African American, and 1 was Cambodian. The median (range) age at diagnosis of NS was 2.4 (1.5–9.7) years. Four patients (50%) had primary steroid resistance; others developed secondary SRNS at a median of 2.6 (range 0.5–6.7) years after diagnosis. Patients were categorized as having secondary SRNS based on their failure to demonstrate any response to 6 weeks of daily prednisolone therapy (2 mg/kg/day) during a relapse. Detailed data for each of the 8 patients is provided in Table 1.

**Table 1:** Clinical and laboratory data in children with SRNS-DTT

Patient	1	2	3	4	5	6	7	8
Steroid resistance	Secondary	Secondary	Primary	Primary	Primary	Primary	Secondary	Secondary
Time to steroid resistance (months)	13	6	–	–	–	–	50	81
Renal biopsy histopathology	MCD	MCD	MCD/IgM	MCD/C1q	FSGS	MCD/mesangial expansion	MCD/IgM	FSGS
Genetic testing	Not done	Not done	Variants of unknown significance	Variants of unknown significance	No mutations	Not done	Not done	No mutations
Immunosuppressive medications other than steroids prior to IV CTX	Tac + MMF	Tac	Tac	Tac	Tac, followed by Rituximab	Tac + MMF	Tac, followed by oral CTX	Tac, followed by cyclosporine
Age at start of CTX (years)	4	3.8	6.5	13.1	10.5	10.5	6	10
Serum albumin at start of CTX (g/dL)	< 1.0	< 1.0	1.3	< 1.0	1.7	2.2	1.8	< 1.0
IV CTX doses	5	6	6	6	6	6	6	4
IV methylprednisolone	With 4th and 5th CTX infusion	None	None	None	None	With each CTX infusion	With each CTX infusion	With each CTX infusion
Remission	CR	CR	NR	PR	NR	CR	CR	NR
Time to remission (months) after last CTX dose	0.5	6.5	–	4.5	–	8	After 4th CTX infusion	–
Immunosuppression after completion of IV CTX at last follow up	MMF	Tac	Tac + MMF	Myfortic	None	MMF + prednisone	None	None
Outcome at last follow up (years after completion of IV CTX)	CR (10.5)	CR (3.7)	Persistently nephrotic; normal renal function	PR (1.3)	ESRD	CR (8.5)	Relapsed (0.9); steroid dependent	ESRD

*MCD* Minimal change disease, *FSGS* Focal segmental glomerulosclerosis*, IV CTX* Intravenous; yclophosphamide, *Tac* tacrolimus, *MMF* Mycophenolate mofetil, *CTX* cyclophosphamide, *CR* Complete emission, *PR* Partial remission, *NR* Non responder, *ESRD* End stage renal disease

Renal histopathology showed minimal change disease (MCD) in 6 patients (75%) (2 classic MCD, 1 MCD with mesangial expansion, 2 MCD with IgM deposits, 1 MCD with C1q deposits) and focal segmental glomerulosclerosis (FSGS) in 2. Immunosuppression was guided by the primary nephrologist and therefore varied based on nephrologist preference and patient/family choice. All patients had failed tacrolimus therapy in conjunction with oral corticosteroids; two children failed dual therapy with tacrolimus and mycophenolate mofetil (MMF). Only one patient received rituximab (patient #5), since the majority of the patients were treated in the era before Rituximab became more commonly used in the setting of NS, especially SRNS; in 1 patient insurance authorization for its use was denied. Four of the eight patients had genetic testing and all were negative for known mutations.

### Treatment with CTX

Informed consent (verbal) was obtained from all patients/families after discussing the pros and cons of CTX therapy. All patients had normal renal function and were treated with monthly infusions of IV CTX; the dose of IV CTX was 500 mg/m2 for the first infusion, 750 mg/m2 for the second infusion and 1000 mg/m2 for the remainder of the infusions; the dose was not adjusted based on the effect of the prior infusion on the white blood cell count. Six of the 8 patients received a total of 6 monthly IV CTX infusions. One patient (patient #1) received only 5 infusions as she went into CR after the fifth infusion (the fourth and fifth infusions were combined with IV MP), and one patient (patient #8) only received 4 doses of IV CTX since he rapidly progressed to ESRD. The dose was adjusted for renal function for patients with deteriorating function. Four patients received concomitant IV MP infusions at a dose of 30 mg/kg (maximum 1 g/dose).

### Outcomes

**Fig. 1:**
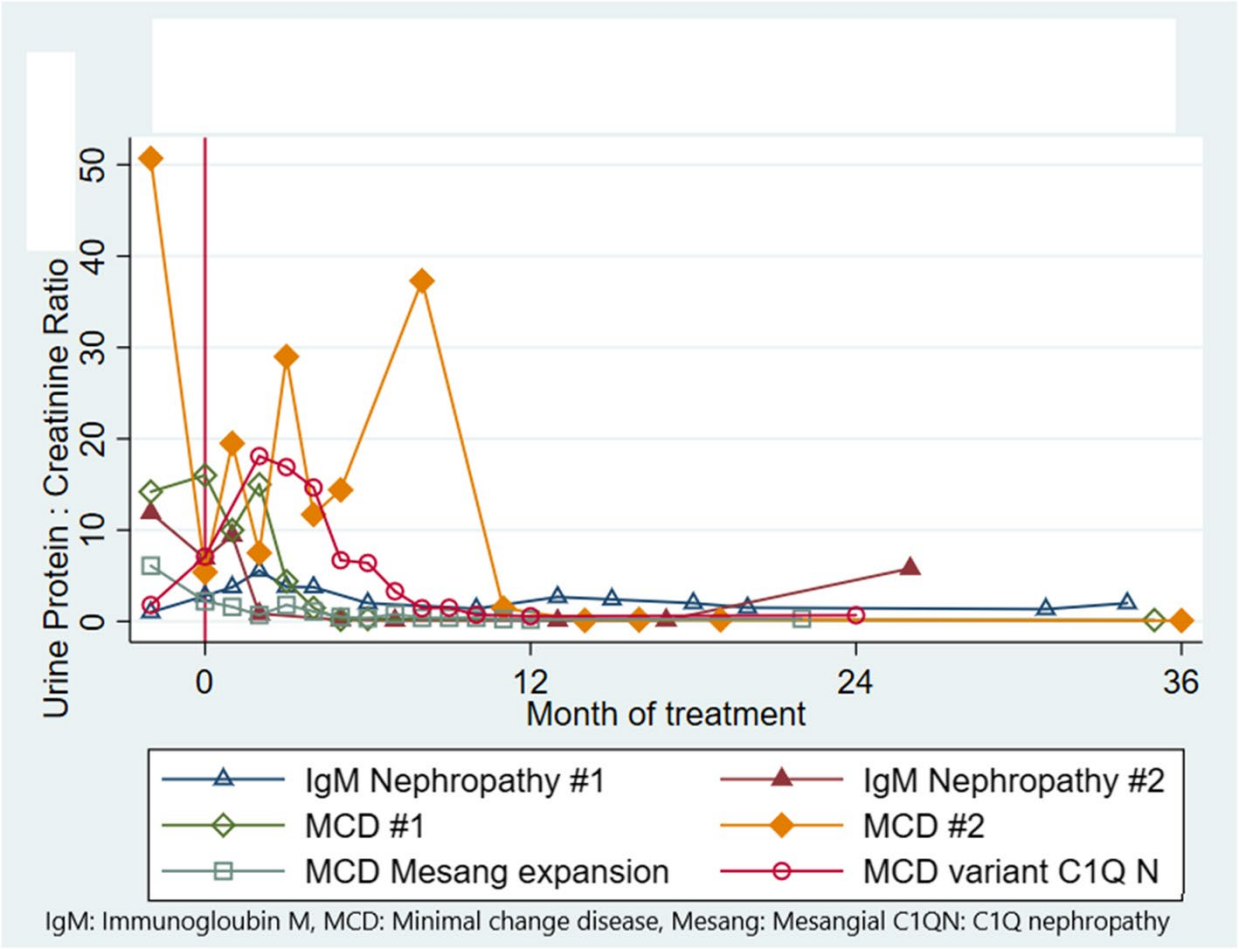
Timeline of the urine protein/creatinine ratio (mg/mg) in the 6 patients with minimal change disease and its variants

Figure 1 depicts the timeline for the 6 patients with MCD and its variants, with respect to IV CTX infusions and the Up/c (in mg/mg). Four patients (50%) went into CR, one patient achieved PR and three patients did not respond. Three of the four responders had MCD on biopsy. Excluding the 1 child who responded after the 4th infusion (patient #7), the median time to CR was 6.5 (range 0.5–8) months after completion of IV CTX infusions. Three patients remained in CR (median 8.5 years; range: 3.7–10.5 years) after completion of CTX; one child relapsed and became steroid-dependent (patient #7). No infections or life-threatening complications related to IV CTX were observed. The child with PR remained in that state 1.7 years after completion of CTX; of the 3 children who were non responders, 2 progressed to ERSD and one has remained nephrotic but edema free and with normal renal function.

After completion of IV CTX, the 4 patients who responded were maintained on MMF, myfortic or tacrolimus (or a combination) for different periods of time. One patient (patient #1) received MMF at 300 mg/m2 per dose twice a day for 2.5 years after which her MMF was discontinued; she remains in remission at last follow up (10.5 years after completion of IV CTX) without any immunosuppressive medications. Patient #2 was maintained on tacrolimus and remains in CR; given the severity of his nephrotic syndrome and the crippling anasarca he had previously experienced, we were very reluctant to try him off tacrolimus. The third patient (patient #4) was maintained on myfortic at a dose ranging from 568 to 675 mg/m2 per day, and is in PR. The last patient (patient #6) was maintained on MMF at a dose ranging from 278 to 362 mg/m2 per dose twice daily, as well as oral prednisolone (5 mg every other day); the prednisolone was eventually tapered off. He remains in CR.

## Discussion

There remains an urgent unmet need to develop new approaches for the treatment of children with SRNS who fail a trial of CNI therapy [3]. While investigators have attempted various combinations of immunosuppressive therapies such as MMF [15], ofatumumab [16] and rituximab [8, 17, 18], none have been effective in sustaining remission in a significant proportion of treated children; moreover, some have been associated with potentially life-threatening complications, not to mention their significant cost [19–21]. All of these factors have limited their widespread adoption for the treatment of SRNS in children, and recommendations for their use are not based on robust evidence [13]. While rituximab has been recommended as the 1st line agent in children with DTT-SRNS (Grade C recommendation), its efficacy in inducing long term remission is low and it is associated with significant potential side effects, is expensive and may need re-dosing when B-cell repopulation occurs [13].

Based on previously referenced data using IV CTX in children of Asian-Indian ethnicity with SRNS, at our center our practice has been to offer this therapy in the setting of SRNS-DTT. IV CTX has also been demonstrated to be of benefit in adults with SRNS; although remission rates are lower compared to CNIs and time to remission longer, unlike patients treated with tacrolimus, CTX was associated with a lower relapse rate after discontinuation of therapy [22] . Our current study explored the risks-benefits of its use in children and demonstrates that IV CTX can be a very effective strategy in a subset of children with SRNS-DTT in North-America: those with MCD on biopsy. Fifty-percent (n 4) of the children treated with IV CTX went into CR that was sustained over a considerable period of time, with preserved renal function, which is a heartening observation. These 4 children represent a 100% of those who had ‘pure’ MCD on biopsy and 50% of those who had MCD variants. Only 1 child with an MCD variant was completely unresponsive and subsequently also failed rituximab therapy- he remains edema free and with normal renal function. In contrast, both children with FSGS failed to show any response and both progressed to ESRD. While we cannot draw definitive conclusions due to our small sample size, it is possible that the concomitant use of IV MP along with IV CTX may be of benefit in attaining remission (or faster time to remission); 3 of the 4 patients who received IV MP went into CR and only 1 child who attained CR had not received IV methylprednisolone (patient #2). Only randomized controlled trials with IV CTX can help tease out whether IV CTX by itself was responsible for inducing remission.

At last follow up, only one of the children who went into CR or PR remains intermittently on steroids; he relapsed after going into CR and has become steroid dependent with normal renal function (patient #5). He too failed rituximab therapy after IV CTX.

Interestingly, the time to remission was quite late (unexpectedly so for us) after start of IV CTX. This is important to realize since failure to respond early on in the course may lead some treating teams to abandon therapy. None of our patients needed hospitalization for any serious illness- no life-threatening infections were noted, nor was hemorrhagic cystitis seen. Transient alopecia was common during the treatment. Clearly longer-term follow-up is needed to assess long term risks such as infertility and malignancies.

Out study is limited by its retrospective nature and small sample size. There was no consistent immunosuppressive protocol that was followed after patients were deemed unresponsive to tacrolimus, and the IV CTX treatment regimen also varied somewhat. Moreover, most of these patients were treated in the era before rituximab become more commonly used in children with SRNS. Lastly, only 4 of the 8 children underwent genetic testing; having stated that, we would like to point out that all 4 children who did not get genetic testing, went into CR. While we recognize that current guidelines do not recommend the use of CTX as first line therapy in children with SRNS, the intent of our study is to highlight that a subset of children with SRNS, who are refractory to all other therapies, may benefit from IV CTX (possibly in conjunction with IV MP); if such children can achieve CR, this would be much more beneficial than having them progress to ESRD.

## Conclusions

In summary, our pilot data demonstrate that IV CTX infusions can be a useful strategy to induce remission in North American children with SRNS-DTT who have MCD or its variants on biopsy. Based on our results, our hope is that other centers will consider such an approach since this has the potential to avoid progression to ESRD and add to the experience with this agent.

## Data Availability

The datasets used and/or analysed during the current study are available from the corresponding author on reasonable request.

## Abbreviations

SRNS: Steroid resistant nephrotic syndrome
CNIs: Calcineurin inhibitors
IV: Intravenous
CTX: Cyclophosphamide
CR: Complete remission
SRNS-DTT: Difficult to treat SRNS
MCD: Minimal change disease
NS: Nephrotic syndrome
ESRD: End stage renal disease
Up/c: Urine protein to creatinine ration
MMF: Mycophenolate mofetil
MP: Methylprednisolone
PR: Partial remission
FSGS: Focal segmental glomerulosclerosis
